# Contextualizing the findings of a systematic review on patient and carer experiences of dementia diagnosis and treatment: a qualitative study

**DOI:** 10.1111/hex.12162

**Published:** 2013-11-28

**Authors:** Frances Bunn, Katie Sworn, Carol Brayne, Steve Iliffe, Louise Robinson, Claire Goodman

**Affiliations:** ^1^ Centre for Research in Primary and Community CareUniversity of HertfordshireHatfieldHertfordshireUK; ^2^ Social Policy Research UnitUniversity of YorkYorkUK; ^3^ Department of Public Health and Primary CareUniversity of CambridgeCambridgeUK; ^4^ Research Department of Primary Care and Population HealthUCL Medical SchoolLondonUK; ^5^ Institute for Ageing and HealthNewcastle UniversityNewcastle upon TyneUK

**Keywords:** dementia, public participation, qualitative, systematic review

## Abstract

**Background:**

Involving service users in the systematic review process is seen as increasingly important. As systematic reviews often include studies from diverse settings and covering a time span of several decades, involving service users in consideration of applicability to specific populations or settings might make reviews more useful to practitioners and policymakers.

**Objectives:**

To test and contextualize the findings of a systematic review of qualitative studies looking at patient and carer experiences of diagnosis and treatment of dementia.

**Methods:**

Results from the systematic review were discussed in focus groups and semi‐structured interviews with patient, public and professional participants in the South East of England. Analysis was guided by coding frameworks developed from the results of the systematic review.

**Participants:**

We recruited 27 participants, including three people with dementia, 12 carers, six service providers and five older people without dementia.

**Results:**

Findings from the focus groups and interviews were consistent with those from the systematic review and suggest that our review findings were applicable to the local setting. We found some evidence that access to information and diagnostic services had improved but, as in the systematic review, post‐diagnosis support was still often experienced as inadequate.

**Conclusions:**

Focus groups and interviews with service users and their representatives can provide useful contextual information. However, such strategies can require considerable investment of the part of the researcher in terms of time and resources, and more work is needed to refine strategies and establish the benefits for patients and the organization of services.

## Introduction

The rationale and use of systematic reviews for evaluating and synthesizing information are well established.^1^, ^2^ However, in recent years, there has been an increasing interest in the way in which systematic reviews are used^3^ with researchers increasingly expected to consider the wider impact and relevance of their work. Involving service users and members of the public in systematic reviews has been seen as one way of improving their quality, relevance and impact,^4^ and there are a growing number of examples of users being involved in systematic reviews.^5^, ^6^, ^7^

A recent study found examples of service user involvement at various stages of the review process including protocol development, review conduct and translation and dissemination of findings.^5^ The latter might be facilitated by involving public and professional groups in contextualizing the results of systematic reviews, and a number of commentators have suggested that considerations of applicability to a local setting or specific population have the potential to make systematic reviews more relevant to policymakers.^8^, ^9^, ^10^

In this paper, we describe our efforts to involve patient, public and professional participants in the contextualization of the findings of a systematic review of qualitative studies looking at patient and carer experiences of diagnosis and treatment of dementia.^11^ Improving diagnosis and treatment of people with dementia is high on the policy agenda in the UK,^12^ and the aim of the review was to inform the debate about early diagnosis and service provision. From the systematic review, we identified key themes relating to patient and carer experiences, barriers and facilitators to diagnosis, and types of support that might be helpful for people newly diagnosed with dementia and their families.

The review included 102 studies from 14 different countries conducted over a 22‐year time period. Despite this diversity, the themes identified were remarkably consistent. There were, however, a range of experiences and views that warranted further analysis, and we were interested in testing and reviewing the findings with user groups and their representatives. The aim of this study was to confirm review findings, assess applicability to the local setting and consider to what extent the review resonates with national and local policy. Whilst user involvement in systematic reviews is not new, there are few previous examples of testing the findings of systematic reviews with user groups in this way.

## Methods

The methods for the systematic review are described elsewhere.^11^ Once data extraction and preliminary analyses were conducted, we held a series of focus groups and interviews with key stakeholders in the local area. Focus groups were our preferred approach as they allow the observation of interaction between participants and provide direct evidence about similarities and differences in opinions and experiences.^13^ However, where necessary, for example due to participants cancelling at short notice, we conducted interviews with individuals or couples instead of focus groups. Focus groups and interviews took place between April and August in 2011.

It has been suggested that systematic reviews might benefit from a range of practitioner, user and community expertise and knowledge.^14^, ^15^ We used a purposive sampling approach to recruit stakeholders with a variety of experiences and knowledge about dementia; this included practitioners who worked in dementia services, voluntary sector representatives, current service users (people with dementia and their carers) and a group of older people without dementia. The rationale for the latter was that including the views of a group without dementia, but who have a societal experience of other people (e.g. friends and peers) receiving a diagnosis and treatment for dementia, would allow for more complete contextualization. The purpose was to discuss the review findings and ascertain participants' views on the initial results of the review. The approach drew on that used by one of the authors in a previous review on the prevention of wandering in dementia.^16^

### Recruitment

Recruitment took place in one geographical area in the South East of England. We used a variety of approaches to recruit participants for focus groups. Older people with dementia were recruited via a consultant psychiatrist from a memory clinic. Once they had expressed an interest in participating, their details were sent to the research team who then contacted them to talk to them in more detail about the study. Family carers of people with dementia were recruited from a support group at a day hospital and via a dementia cafe run by the Alzheimer's Society. Both groups provided people with dementia and their carers a chance to socialize and discuss relevant issues, with peer support for carers an important function of both. Older people without dementia were recruited via an older people's community group that focused on self‐managed education and learning. Members of the research team attended meetings of these groups to give a brief presentation about the study. People expressing an interest in participating were given an information sheet and had the opportunity to discuss the study further. Voluntary service providers were recruited via previously established links with local voluntary organizations, such as the Alzheimer's Society and county‐wide carers organization, and statutory service providers were identified through links with the local older people's mental health services.

### Focus group procedures

There are a number of issues that need to be carefully considered when planning focus groups with people with dementia including procedures for consent and assessment of capacity, the potentially distressing effect of addressing sensitive issues and the importance of familiar surroundings.^17^, ^18^ For people with dementia, careful consideration was given to the consent process and assessment of capacity. Before the focus group began, the researchers explained the purpose of the study, checking that the participants understood and were able to communicate their decision either verbally or in writing. Participants were informed that they could have a break or withdraw from the discussion at any time. In addition during data collection, researchers made every effort to detect non‐verbal signs of distress or indication they wished to withdraw. Participants were given a £10 voucher in appreciation of their time, and their travel expenses were reimbursed.

Although the groups for carers and service providers included up to eight participants, the group involving people with dementia was limited to no more than three participants with dementia. Locations for focus groups were chosen to facilitate access and minimize inconvenience or potential distress for participants, with groups for people with dementia and carers held at locations with which participants were already familiar. People with dementia were offered the opportunity to have a family member or friend to participate with them in focus groups or interviews. Focus groups were facilitated by academic staff on the study team (FB & KS) who were introduced to the participants as researchers. Groups were taped and transcribed in full, and another researcher (EM) took additional notes.

Topic areas for discussion were informed by the themes that emerged from our systematic review. The format of the group was tailored to the participants. For example, groups with service providers and older people without dementia began with a presentation of key findings from the review, and these were used as the basis for the subsequent discussion. For carers and people with dementia, results from the study were presented in a more informal way but were still used as a guide for the discussion, for example, by presenting them as common issues that people newly diagnosed with dementia might experience. Focus groups and prompts focused particularly on barriers and facilitators to diagnosis and issues around service delivery. The duration of the interviews was 30–50 min, and the focus groups were between 40 and 90 min.

### Analysis

As the purpose of the study was to contextualize the findings of our systematic review, we did not look for new themes but rather looked to see whether data from the review could translate into local or current experience. The results of the thematic analysis from the systematic review were used to develop coding frameworks for analysis of the focus group transcripts. Transcripts were then imported into Nvivo and coded, using the pre‐defined coding framework. Initial coding was done by one researcher and checked by a second. Any disagreements were resolved by discussion.

## Findings

### Characteristics of participants

We conducted four focus groups and three interviews with a total of 27 participants (three people with dementia, 12 carers, six dementia service providers and five older people without dementia). All the people with dementia had received a diagnosis and had mild or moderate dementia. We had originally intended to hold an additional focus group with people from black and minority ethnic groups. However, despite networking with a number of local service providers and voluntary organizations, we were unable to recruit people with dementia or carers from any BME groups. Further details of participants can be seen in Table 1.

**Table 1:** Details of focus group participants

Identifying Number	Type of participants	Number of participants	Location of focus group
FG 1	Carers	1 Focus group (FG) with 8 participants (5 male, 3 female)	Dementia cafe run by Alzheimer's Society
Interview 1 and interview 2	Carers	2 Interviews (2 female carers)	Day hospital (ran support services for patients and carers)
FG2	PWD (and their carers)	1 Focus group (FG) with 2 PWD (one male, one female) and their carers (1 spouse, 1 adult daughter)	Day hospital (ran support services for patients and carers)
Int 3	PWD (and their carer)	1 Couple interview (male with dementia + spouse)	Day hospital (ran support services for patients and carers)
FG 3	Service providers	1 Focus group with 6 participants (3 female, 3 male) Included 1 county council commissioning manager, two representatives from voluntary organizations, 3 health‐care professionals working with people with mental health problems including dementia	University
FG 4	Older people without dementia	1 Focus group with 5 participants (3 female, 2 male) – recruited via local community group	University

#### Confirmation of key themes from the review

**Figure 1:**
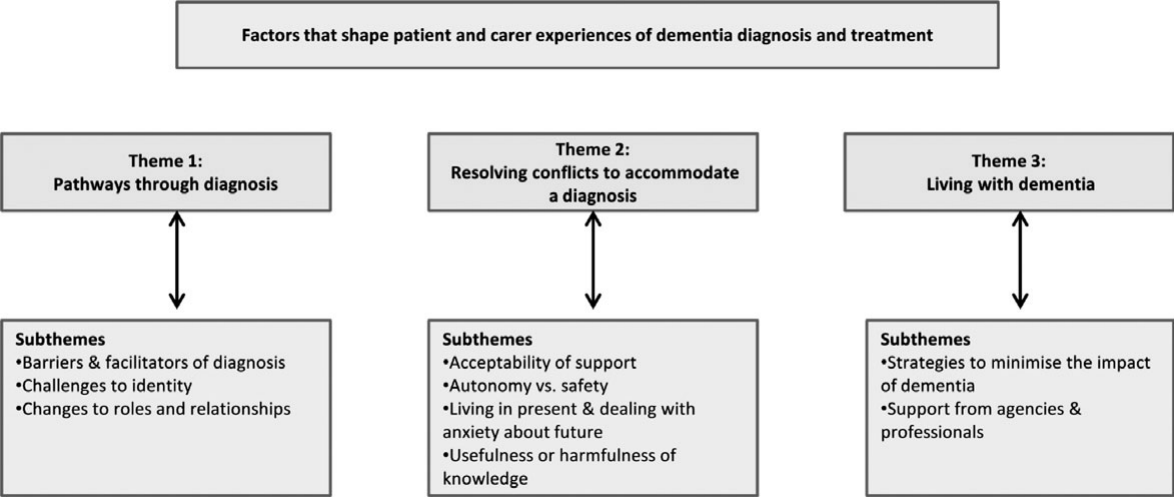
Themes and subthemes. This figure shows the three overarching themes and the related subthemes that emerged from the systematic review.

The systematic review identified three overarching thematic categories, which describe a process of diagnostic transition. These were 1) pathways through diagnosis, 2) conflicts that need to be resolved to accommodate the diagnosis and 3) living with dementia. Themes and subthemes can be seen in Fig. 1 and are described in greater detail elsewhere.^11^

Findings from the focus groups and interviews substantiated those of the systematic review (see Table 2).

**Table 2:** Cross‐analysis of themes from the systematic review and findings from the focus groups and interviews

Subthemes	Supporting evidence from focus Groups/interviews	Quotes from interviews and focus groups illustrating the themes
Theme 1: Pathways through diagnosis		
Barriers and facilitators to diagnosis	Stigma, normalization of symptoms, lack of awareness identified by all groups of participants as barriers to diagnosis. Some saw dementia as worse than physical illness. For some gradual awareness something wrong – for others trigger event such as fall or bereavement led to help seeking. In some instances, another family member (e.g. son or daughter) recognized problem before PWD or their spouse	‘Alzheimer's and dementia does have a stigmatisation to it and some people don't want that diagnosis’ (*Service provider*) ‘it's the worst thing any of us want to be told, to lose your personality is appalling’ (*Older person without dementia*) ‘dementia starts and you are not really aware of it’ (*Carer*)
Challenges to identity	All groups aware of the impact of dementia on identity. PWD tried to maintain identity through activities and carers attempted to reinforce identities positively by focusing on PWD abilities rather than mistakes or lack of recall. Symptoms of dementia made some people withdraw from previous activities (although they often re‐engaged later)	‘I could fix cars and everything you know, but it's all gone’ (*PWD*) ‘and there's a fear of not being in control of yourself’ (*Service Provider*) ‘I do get out now, but I didn't want to then’ (*PWD* referring to when first diagnosed)
Changes to roles and relationships	All groups aware of changes to roles & relationships, and the increased burden on the carer PWD and carer had to adjust to increasingly unequal relationship. PWD and carer may interpret things differently but meaning is often negotiated jointly Social networks change	‘all the changes you need to make to yourself and your own behaviour in order to deal with this problem’ (*Carer*) ‘there are a lot of people, friends and so forth who gradually move away you know.. all they want me to say is ‘we are coping alright’ (*Carer*)
Theme 2: Resolving conflicts to accommodate a diagnosis		
Acceptability of support	The decision to accept support depended on the stage of the illness, interfamily support, readiness to accept a diagnosis and appropriateness of services. Services might be rejected by carers or the PWD	‘he wouldn't get on transport… he never accepted that he was the one that needed the help’ (*Carer*) ‘I don't want to put things in practice that might not suit him (*Carer*)
Autonomy vs. safety	Issues around autonomy and risk came through particularly amongst people with dementia and their carers. Aspects of dependence were unavoidable, and sometimes it was impossible for carers to enable PWD to maintain skills associated with sense of self, for example driving.	‘you can be over‐protective, I think sometimes (Carer) ‘I walk out the town and back every day and she (referring to her daughter) stopped it because it's six miles there and back. She said it's too far, so I got stopped’ (*PWD focus group*)
Living in present and dealing with anxiety about future	PWD and carers tended to focus on day‐to‐day living but were also dealing with fear about the future. Fears included getting worse, not coping, having to put PWD in nursing home	‘I mean if she got any worse we are going to be in a right mess.. I'll be forgetting things and if she can't do things’ (*PWD* referring to carers physical health)
Usefulness of harmfulness of knowledge	All groups acknowledged the importance of information but readiness to receive information clearly varied between participants.	‘They printed off some information about Alzheimer's which I read but I think you said you didn't want to bother, you thought, oh, well’ (carer referring to partner with dementia) ‘I don't think people want to know about it, they put it on the back burner until it actually hits’ (*Older person without dementia*)
Theme 3: Living with dementia		
Strategies to minimize the impact of dementia	The use of strategies to cope with the impact of dementia was clear in the interviews with PWD and their carers. Included emotional strategies (e.g. humour, finding meaning and joy) and social strategies (e.g. relying on family support and adapting social networks) Minimization of losses feature of early stages of dementia – resilience displayed in adapting and sustaining routines Emphasis on trying to be normal (staying active, downplaying symptoms, retaining skills)	‘and we joke about it’ (Carer) ‘I can go with the children if they're doing something from school’ (Carer referring to grandchildren)
Support from agencies and professionals	People reported both positive and negative experiences of interactions with health‐care professionals. All groups suggested that support, particularly post‐diagnosis, was lacking. Both service providers and carers referred to developments in local service provision designed to improve diagnosis and treatment.	‘the memory clinic.. the only positive thing I can say is that we had a very lovely doctor’ (Carer) ‘nobody tells you how to care (carer) ‘the situation now for someone getting a diagnosis should be very different because this year a programme was being rolled out to support people in those early stages’ (Carer)

##### Theme 1: Pathways through diagnosis

Persistent barriers to early diagnosis identified in the systematic review were stigma, the normalization of symptoms and a lack of awareness about the signs and symptoms of dementia. This was substantiated in all the interviews, and focus groups with symptoms often normalized as part of the ageing process:

He was forgetting things but you put it all down to ‘oh, that's my age’, sort of thing don't you? I hadn't really thought seriously about it.

Carer FG1

The theme of stigma was reinforced by participants and further illustrated by accounts of attempts to conceal memory problems and how carers colluded with their spouse to hide their symptoms.

There is a long period hiding and then you collude with the hiding because it's not yours to share and that's part of the problem.

Carer FG 1

Findings from the review suggested that doctors being slow to recognize symptoms or reluctant to give a diagnosis could also be a barrier, and this was confirmed in our focus groups. One service provider said:

in most of the cases where I've seen there's been a long delay between them first recognising a symptom, and receiving a diagnosis. I think the GP was the main sort of blocking point there.

Service provider FG 3

However, a number of participants in this study had found their GPs helpful and did not encounter problems being referred to memory services.

A focus group with older people who did not have dementia demonstrated a range of viewpoints and levels of knowledge, which appeared to be partly dependent on previous proximity to a person with dementia. As a group, they had an awareness of dementia as a growing societal problem and some knowledge of different types of dementia and related risk factors. However, they had less knowledge of available services and had very negative attitudes towards dementia:

most people I know would rather have cancer than Alzheimer's.

Older person FG 4

##### The impact of diagnosis

A recurring theme throughout the literature concerned the impact of dementia on identity and roles and relationships, both for the person with dementia and for carers. This was confirmed in the focus groups and interviews with people with dementia giving specific examples of changes in their roles and responsibilities, for example no longer being able to prepare meals, maintain their car or deal with financial matters. Often, the carer had taken on these responsibilities. One carer referred to the increasingly unequal nature of their relationship with the person with dementia although they did this in such a way as to try and reinforce the identity of the PWD by referring to their past abilities.

I mean maybe it was 75% (refers to the PWD) ‐ doing things years ago when now it's more 50–50 or even a bit further down towards me, but as I said before it was 75% you doing things before wasn't it.

Carer Int 3

There was evidence from both the review and the focus groups that carers focused on the abilities of the person with dementia rather than their mistakes or lack of recall. Even when it became impossible for people with dementia to maintain certain activities, such as driving, carers sought to frame this in a way that was less distressing. For example, when discussing giving up driving, one carer said to his wife:

I think it is not a fact you can't drive. Probably if I was with you, you could. The problem is, as the doctor explained, if there was a slight accident and it wasn't your fault, the insurance company would find any reason whatsoever not to pay.

Carer FG 2

Participants also talked about how relationships with friends and wider social networks had changed. There was evidence that people withdraw from activities (either temporarily or permanently) they once enjoyed because they were worried of what other people will think or because they were no longer able to cope with them. The potential to become socially isolated came out particularly strongly from the focus group held with carers at a local dementia cafe, many of whom had been carers for some years:

there are a lot of people, friends and so forth who gradually move away you know…all they want me to say is “we are coping alright”

Carer FG 1

However, there was also evidence that people did adapt to their changing situation and managed to maintain or create new, social contacts, although this was often achieved by altering expectations and activities. For example, one carer said she now invited friends round for tea and biscuits rather than a meal and one couple who, since the wife's diagnosis, had begun going to listen to live music in pubs through which they had developed a new circle of friends.

##### Theme 2: Resolving conflicts

From the systematic review, it was clear that a number of tensions existed as people struggled to accommodate a diagnosis of dementia and preserve a sense of self in the face of increasing symptoms. This was also evident in our study with some participants in the early stages appearing to resist a diagnosis. One carer said of her mother

she's in denial a little bit.

Carer FG 1

As with the systematic review, we found evidence of people adopting a variety of attitudes towards dementia. Mind‐sets were often mirrored by the PWD and their carer, and both had found a common way to attach meaning to previous symptoms, the future (e.g. hope for improvement) and the loss of everyday skills such as driving. Although some people thought about the potentially devastating impact that dementia could have on their future the emphasis tended to be on the present. This was reflected in attitudes towards information. Although one recently diagnosed man spoke of going on the internet to find information relating to the life expectancy of people with dementia others in the early stages rejected or resisted information. For example, one couple who had recently received a diagnosis were not ready to learn more about the condition or its prognosis. When discussing information they had been given at the memory clinic the carer said:

I haven't bothered to read it, to be honest. I think as ___ is at the moment, we just leave it at that. If it gets worse, then I'll have a read and see if there's a reason why, and then we'll contact the GP again. And I'm not one for reading medical literature too much.

Carer FG 2

However, for some people, a poor understanding of what their diagnosis meant could lead to confusion.

I mean, as the lady said, it could be dementia, it could be Alzheimer's. Well, there's a vast difference between the two, a vast difference. I mean, Alzheimer's is a nasty illness whereas dementia can be handled quite easily, and we all get dementia, I suppose, at some time or other.

Carer FG2

The participants we spoke to were at different stages in the dementia trajectory and those in the earlier stages of the disease had not necessarily accessed services. People had not accessed services, because they were unaware of what was available, because services did not meet their needs or because they were not yet at the stage where services were required. In some instances, there was a tension between the needs of the carers and the wishes of the person with dementia. For example, one carer spoke of how her husband refused to stay at a dementia lunch group unless she remained with him and another talked about her husband refusing to have carers in the house:

No, I thought “I'll see how he goes, I don't want to put things in practice that might not suit him, I want to see how far he will progress and then take up whatever it is.” I mean, when the carers came and he wouldn't have them in the house, you think “Fine, that's one thing we can't do again”.

Carer interview 1

##### Theme 3: Living with dementia

The review highlighted how people with dementia and their families often adopted strategies to manage the disease, minimize losses, reduce social isolation and maintain normalcy. As already noted, these strategies were also apparent in the interviews and focus groups. Carers attempted to reinforce the identity of the person with dementia, facilitate their participation in social events and activities and help them to maintain a sense of their former self. There was evidence that identity was maintained through activities, although the extent of participation might lessen (e.g. going to watch rather than play bowls). It was also clear that coping strategies changed to reflect the stage of the illness and the fluctuating nature of the disease.

#### Supporting people with dementia and their carers

##### Diagnosis

Although many of the people with dementia and their carers we spoke to reported positive experiences of their interactions with their GPs and other health‐care professionals, some had been less fortunate. One male carer said that when his wife was diagnosed at a memory clinic, it was ‘the cruellest experience of his life’. Even where communication had been good, the overall experience of diagnosis was still traumatic. Service providers in our focus group acknowledged that, in their experience, most memory clinics were currently held in environments that may be frightening or shocking for patients and carers.

##### Post‐diagnosis support

Problems identified in the review included a lack of information and specialist services, and inadequate support for carers. Participants confirmed this as their experience although there was some evidence to suggest that post‐diagnosis support and information provision were improving in the local area. Support services and voluntary organizations were not always well signposted, and it sometimes took a while for people to access informal support such as that offered by the Alzheimer's Society.

And one's in a state of shock in the beginning you don't actually function, you don't use all of your normal strengths and it was 2 years before I decided to walk into the Alzheimer's Soc to see if there was some sort of support.

Carer FG 1

Although when people did make contact with local voluntary organizations, they found them very helpful, it was also frustrating for carers that there was not a central repository of information. One carer remarked:

there is not one person for example or one organisation where the specialist or even your GP can say “contact that person and they will tell you what's available”

Carer FG 1

As in the systematic review, we found that much of care for people with dementia was being provided by family members, in particular spouses. Carers highlighted the constant vigilance and care required and the huge impact this has on their own lives:

I mean it's really 24 hours a day.

Carer FG 1

One woman commented on how even when her husband was at a day centre she had little time to relax

That's supposed to be my day off, when I've got five hours leisure,…and what do you do? You rush home, you strip his bed, and you get everything washed, go to the shop, you get the shopping in, and you've got an hour left perhaps to relax, and then I'll come and get him

Carer Int 1

#### Information provision

The review identified polarized views about needs for information about dementia, and this was reinforced in the interviews and focus groups. It was clear that greater thought needed to be given to how to organize and provide information that is responsive to an individual's interests and priorities and differentiates between the needs of the person with dementia and their carer. The focus group with service providers suggested that they were aware of this and were using different approaches to information giving.

And it's about retrieving information as and when appropriate…. it can be tailored individually to that person because you get some people that like to have…the information and..can comprehend it and you know and might not be contact for a month or two but there will be other people where you need to see every other week to drip feed the information and to support them at their level and at what pace they are…

Service provider FG 4

Although attitudes towards information about prognosis differed, it appeared that most service users would benefit from early information about benefits and entitlements. One male carer remarked that there was ‘*a lot out there to help you but you don't find out’*. However, information provision alone was not enough. One man said that he and his wife had been ‘inundated with information’ but that what he most wanted was respite care so that he could go and play golf.

#### Peer support

Findings from the systematic review suggested that attitudes towards activities that enabled people with dementia and their carers to meet with their peers were largely positive. However, it was clear that it could be stressful or distressing to see people with more severe symptoms. From our small sample, it appeared that peer support was particularly beneficial to carers. Referring to the support group at the day hospital, one carer said:

It's just a chat and have a laugh, cup of coffee and talk to whoever's next to you, but if someone says ‘so and so did this and I don't know what to do’ then everybody puts their tuppence worth in and holds them up in effect and supports them as much as they can, but that two hours is very nice indeed

Carer Int 1

One female carer said that the dementia cafe support group (run by the Alzheimer's Society) had totally altered her life and another said it had provided the emotional support he had not been able to get elsewhere.

## Discussion

Focus groups and interviews conducted with a local sample of patient, public and professional participants substantiated the themes that had arisen in the systematic review, demonstrating that the findings from the review were both current and applicable to a local setting. Experiences of diagnosis identified in the literature were reflected in our study and stigma; normalization of symptoms and a lack of awareness continued to be barriers to diagnosis. Negative attitudes towards dementia were apparent in all patient and public groups but were reflected particularly strongly in the group of older people without dementia. It was also clear from both the review and our sample that dementia represents an enormous challenge to a person's sense of self and that people with dementia undergo a profound transition from a pre‐ to post‐dementia identity. The negative attitudes held towards dementia and the likely emotional impact of a diagnosis make it understandable that professionals approach a diagnosis of dementia with some trepidation.

One of the key themes in the systematic review related to the practical strategies that people adopted to enable them to live with dementia and the support from professionals and agencies. We found similar strategies in our sample, and as in the review, there was evidence that many people showed great resilience as they adapted to the impact of dementia. As in the review, experiences of services varied, although there was some evidence in our sample of improvements in awareness of issues around diagnosis and in signposting to services. However, many of our participants, like those in the review, highlighted the paucity of post‐diagnosis support. Peer support seemed valued, particularly by carers. However, our sample of carers was skewed towards those who were members of peer support groups, and therefore, caution needs to be taken when extrapolating findings to other groups. Moreover, many had been carers for some time, and it is less clear whether peer support is helpful for family carers in the period of transition immediately after a dementia diagnosis. The participants in our focus groups and interviews were at different stages of the trajectory of the illness, and this was reflected not only in their service experiences but also in their knowledge and understanding. Attitudes towards information varied greatly with some wanting specific information about prognosis and others reluctant to find out more about what the future might hold.

In the systematic review,^11^ we highlighted the substantial body of qualitative work relating to the experiences of community‐dwelling individuals with cognitive impairment and their family carers, particularly in relation to the transition to becoming a person with dementia. The focus groups and interviews identified similar findings about the impact of receiving a diagnosis. What this study did reveal was that was a need to know more about strategies that worked, to understand how information is used (or not), how some people engage well with dementia‐specific support and others do not and how factors such as the stage of the illness and levels of interfamily and community support impact on service needs. It was encouraging that there was evidence of greater access to information and diagnostic services although disappointing that several years since the introduction of the national dementia strategy in the UK post‐diagnosis support was still often experienced as inadequate.

The study also raised some fundamental questions about how what we already know, about living with a long‐term condition and being a carer, can help and support the experience of becoming a person with dementia. Many of the issues that arose from the review, for example changes to relationships, caregiver strain and lack of appropriate support and services, are also applicable to other long‐term conditions.^19^, ^20^ However, whilst there is a need to consider what can be learnt from other conditions, evidence suggests that caregiver burden may be greater for those caring for a person with dementia. Changes to roles and increasingly unequal relationships may exacerbate carer stress^21^ as may the behavioural disturbances associated with dementia.^22^, ^23^ Services to support people with dementia need to be tailored to their specific needs. Moreover, even though service needs may be low in the early stages of the illness, it is still important that patients and their carers have contact points that they can return to when needed.

There is increasing interest in the impact of research. It has been suggested that systematic reviews might have greater influence on local policy and practice if the applicability of results to the local setting is considered. This may be particularly important in this instance as UK Government policy initiatives^12^ have meant that dementia services both nationally and locally have been changing. Such changes may mean that results from studies conducted in the past or in different settings may not be relevant. However, the focus groups and interviews confirmed that whilst there appeared to be some improvements in attitudes and service provision, many of the issues identified in the literature have persisted over time and remain pertinent to the local setting.

### The role of stakeholders in contextualizing reviews

There were a number of benefits to the approach we adopted. It allowed us to contextualize our review findings, ensured that the results were grounded in everyday practice, provided a different lens to examine the data and allowed us to compare the findings of the review with our focus groups and interviews. Indeed, the involvement of service users and practitioners allowed us a more nuanced understanding of the systematic review data. However, it should be noted that there are resource implications for a study of this kind. Previous work has suggested that identifying and recruiting participants to focus groups may be particularly problematic for vulnerable groups,^24^ including those with dementia,^25^ and we found this to be the case. It took a significant amount of time to develop the necessary networks to facilitate recruitment and to undertake the focus groups and interviews.

An additional challenge for the researchers was to communicate clearly to participants the purpose of both the review and of the focus groups and interviews. The lay participants in our study did not have any previous knowledge or experience of systematic reviews and were unfamiliar with the concept of user involvement in research. A previous study found that the recruitment of vulnerable older people, and the discussion of emergent findings with study participants, was facilitated by the involvement of experienced public involvement representatives.^26^ It is possible that this strategy would have been useful in our study.

Researchers intending to consult lay participants about the findings of a systematic review need to ensure review teams include people with the necessary interactional and group facilitation skills as well as research skills for collecting and analysing people views.^14^ However, such processes can be costly and can mitigate against the rapid delivery of reviews. Moreover, as there are few formal evaluation of strategies to increase the impact of systematic reviews^27^ or of the effect of involving consumers in systematic reviews,^5^ the benefits of investing resources in this way are not clearly established.

## Strengths and limitations

We found great concordance between the findings of the systematic review and the focus groups and interviews in this study. However, the researchers facilitating the focus groups had also conducted the systematic review, and it is possible that they unconsciously led or interpreted discussions in ways that validated the review. Moreover, the agreement may in part be because our sample reflects the same selection biases found in many of the studies in the review. Analysis of the characteristics of participants in the systematic review suggested that there was a skew towards more affluent, educated populations most of whom were white and lived with a carer. The participants in our focus groups and interviews may also reflect this bias. They had all accessed services; none of our participants with dementia lived alone, and all the people with dementia and their carers were white. However, we took a purposive sampling approach to get a range of experiences and opinions to capture the different types of knowledge (organizational, practitioner and user) which may be beneficial when attempting to understand and contextualize review findings.^15^

## Conclusions

It has been suggested that systematic reviews might be made more relevant to a local setting by involving service users and their representatives in contextualizing and confirming the results. Focus groups and interviews with patient, professional and public participants gave us valuable contextual information and allowed us to substantiate the results of our review. However, it is clear that such strategies can require considerable investment of the part of the researcher in terms of time and resources, particular when involving vulnerable groups such as people with dementia. Further work is needed to refine strategies for service user involvement in the contextualization of results and to establish the benefits.

## Funding

This article presents independent research commissioned by the UK National Institute for Health Research (NIHR) under Research for Patient Benefit (Grant Reference Number PB‐PG‐0808‐16024). The views expressed in this paper are those of the authors and not necessarily those of the NHS, the NIHR or the Department of Health. The sponsor of the study had no role in study design, data analysis, data interpretation or writing of the report.

## Author contribution

F Bunn wrote the protocol for the study, undertook data collection and analysis and wrote the paper. K Sworn undertook data collection and analysis and commented on the paper. C Goodman, participated in study design, provided guidance on the analysis and critically reviewed the paper. S Iliffe, C Brayne and L Robinson participated in study design, interpretation of the results and critical review of the paper. All authors read and reviewed the final version before submission.

## Ethical approval

Ethical approval was obtained from NRES Committee East of England. REC reference 10/H0302/19.

## Conflict of interest

LR received an honorarium from Pfizer for an expert lecture. We have no other conflict of interests.
